# A Single Dose of Fat-Based Energy Supplement to Light Birth Weight Pigs Shortly After Birth Does Not Increase Their Survival and Growth

**DOI:** 10.3390/ani9050227

**Published:** 2019-05-09

**Authors:** Océane Schmitt, Emma M. Baxter, Peadar G. Lawlor, Laura A. Boyle, Keelin O’Driscoll

**Affiliations:** 1 Pig Development Department, Teagasc Animal and Grassland Research and Innovation Centre, Moorepark, Fermoy, Co. Cork P61 NP77, Ireland; peadar.lawlor@teagasc.ie (P.G.L.); laura.boyle@teagasc.ie (L.B.); keelin.odriscoll@teagasc.ie (K.O.); 2 Department of Animal Production, Easter Bush Veterinary Centre, Royal (Dick) School of Veterinary Studies, The University of Edinburgh, Easter Bush Campus, Midlothian EH25 9RG, UK; 3 Animal Behaviour and Welfare Team, Animal and Veterinary Sciences Research Group, Scotland’s Rural College (SRUC), West Mains Road, Edinburgh EH9 3JG, UK; emma.baxter@sruc.ac.uk

**Keywords:** pig, energy, low birth weight, survival, blood glucose

## Abstract

**Simple Summary:**

In modern piggeries, due to the increase in litter size (number of piglets born alive), the number of piglets born at a low birth weight (typically under 1.0 kg) is increasing. Those piglets have a lower chance of survival because of their lower body energy reserves, and therefore are of concern for the farmers. Piglets weighing less than 1.1 kg at birth were given an oral dose of fat-based energy (2 mL of coconut oil or 2 mL of a commercial product), 2 mL of water or were only handled but given nothing. This was done to investigate the effects of providing an energy boost at birth on the chances of survival of small piglets. Parameters measured to assess the piglets’ vitality were survival, blood glucose content, rectal temperature, behaviour test of vigour and weight gain. Unfortunately, there was no effect of the energy dose on the parameters measured. Therefore, we conclude that a single dose of energy at birth does not enhance the chances of survival of small piglets. Therefore, using energy product to improve piglet survival at birth may not be the most efficient strategy.

**Abstract:**

Low birth weight piglets are at high risk of mortality, because of the rapid depletion of their energy reserves after birth. At 3 h postpartum, 405 piglets weighing <1.1 kg were either dosed orally with 2 mL of (1) coconut oil (CO, 74 kJ/2 mL, *n* = 107 piglets), (2) commercial product (CP, 71 kJ/2 mL, *n* = 101 piglets), (3) water (W, 0 kJ/2 mL, *n* = 100 piglets) or (4) were sham-dosed (S, *n* = 97 piglets). Treatments were applied within litter (97 sows). Before treatment piglets were weighed, scored for vitality and blood glucose concentration (subset: CO = 45 piglets, CP = 38 piglets, W = 49 piglets and S = 44 piglets) and rectal temperature were measured. Rectal temperature was remeasured 1 h post-treatment (4 h postpartum). At 24 h post-treatment (27 h postpartum), vitality, weight and blood glucose were remeasured. Piglets were weighed on D5, D7, D10, D14, D21 and at weaning (27 ± 0.1 day old). Mortality rate and cause were recorded until 24h period post-treatment and until weaning. Data were analysed using Generalised Linear Mixed Models in SAS. There was no overall effect of treatment on any of the parameters measured. In conclusion, a single oral of fat-based energy supplement dose at birth did not improve growth, survival, rectal temperature or vitality of low birth weight piglets.

## 1. Introduction

Piglets are born immunologically naive and with low energy reserves. Nevertheless, the first few hours of their life are very energy demanding as piglets have to thermoregulate, dry off, move around the pen to locate the udder and, finally, compete with siblings for acquisition of a functional teat. Neonatal piglets mainly use body reserves of lipids, and then carbohydrates as sources of energy for the production of heat; depletion of fat reserves increases carbohydrates reserves’ consumption rate [1]. Lipid reserves of neonatal piglets seems to be influenced by their birth weight, which reduces the available energy obtained from fat from 175 kJ/kg of body weight (normal birth weight piglets) to 19.5 kJ/kg of body weight (intra-uterine growth retardation (IUGR) piglets) [1], whereas glycogen reserves are not (252 kJ/kg of body weight for all piglets [1]). The only exogenous source of energy for neonatal piglets is colostrum. This contains long-chain triglycerides (LCT), composed of long-chain fatty acids (i.e., LCFA), which account for 40 to 60% of total energy value [2]. However, since colostrum yield does not increase with increased litter size [3], competition for its acquisition is intense in large litters. Thus, this important exogenous energy resource can be monopolised by the early-born and the most vigorous piglets.

Piglets from large litters (i.e., >14 piglets born alive) tend to be born lighter [1] compared to those from smaller litters, and often suffer Intra-Uterine Growth Retardation (IUGR). Although definitions vary between studies, there seems to be general agreement that piglets with a birth weight lower than the last quartile (usually <1.0 kg) are classified as “low birth weight” [4,5] and are known to have a greater risk of dying [6]. However, a distinction should be made between piglets that have a normal allometry, i.e., “small for gestational age”, and those which are disproportional, i.e., IUGR, as the survivability of the latter is lower [7]. For instance, IUGR piglets have similar glycogen reserves but lower lipid reserves than normal piglets and therefore are at greater risk of body reserve depletion if they do not ingest colostrum soon after birth [1]. This exacerbates the risk of mortality through the chilling–starvation–overlying–disease complex [8]. At 18–26 °C, a normal piglet’s body lipids enable a sustained production of heat for ~15 h, while the body reserves in IUGR piglets only enable heat production for 3 h [1]. It is not surprising that neonatal mortality in the pig is more frequently caused by failure to acquire sufficient energy, in the form of colostrum/milk, than by their failure to acquire sufficient amounts of immunoglobulins [9]. This is supported by two experiments by Muns and colleagues, whereby energy supplementation (i.e., without immune material) had similar effects on survival and growth to that of colostrum supplementation [10,11]. Therefore, low birth weight piglets are best targeted by nutritional interventions

Oral energy supplementation after birth is a potential approach to improve the survival of low birth weight (and possibly growth retarded) piglets. Supplementation interventions are not supposed to represent a full meal for the dosed piglets, but rather a boost of energy to be able to reach the udder and ingest colostrum. As lipids are the most important source of energy for neonatal piglets, commercial energy supplements are mostly fat-based, either using LCFA or medium-chain fatty acids (i.e., MCFA) in their formulation. Both LCFA and MCFA enhance the thermoregulatory abilities of piglets similarly [12], but MCFA might be more effective at supplying energy to piglets: the oxidation rate of MCFAs is faster than that of LCFAs and therefore can cover a greater part of piglets’ energy expenditures. For instance, at peak utilisation, MCFA met 35% of piglets’ energy expenditures, while LCFA only met 9% [13]. Thus, the energy needs of colostrum-deprived piglets could be sustained for longer if they are provided with a supplement rich in MCFAs, rather than rich in LCFAs (e.g., 5.8 h vs. 1.2 h, respectively [13]). Medium-chain triglycerides (MCT), composed of MCFA, were found to be more effective than colostrum in increasing plasma glucose of neonatal fasting piglets (both normal and low birth weight), but did not increase the survival rate of low birth weight piglets [14]. In a subsequent experiment, MCT supplementation (25 mL twice in 24 h) to suckling piglets resulted in less active piglets and lower plasma glucose concentration both 30 h and 21 days postpartum, compared to saline solution supplementation [14]. It is possible that such a large dose of MCT gave the piglets a feeling of satiety, thereby disrupted normal nursing patterns [14,15]. Moreover, a 3 mL dose of MCT was found to be sufficient to reduce the mortality rate of piglets born under 1.25 kg to the level of piglets born over 1.25 kg [16]. 

Two recent studies examined the effects of oral supplementation with commercial energy supplements to neonatal piglets of different birth weights, and both found that these supplements were effective in promoting survival in small piglets [5,11]; even if small amounts were used (3 g in Declerck et al. [5]; 3 mL in Muns et al. [11]). However, the definition of “low birth weight” piglets varied between the two studies (birth weight <1.30 kg in Muns et al. [11] and <1.20 kg in Declerck et al. [5]), since it was based on within-litter weight ranking, and treatments were applied to individual litters and not to individual piglets within the same litter. The first aim of the present study was to investigate the effects of neonatal oral supplementation on the survival, growth and indicators of vitality of low birth weight piglets (<1.1 kg), and the second aim was to compare a basic energy source (i.e., coconut oil) with a more complex commercial product (composed of coconut oil, soya oil and added fatty acids). In order to accurately identify treatment differences, the effect of litter was controlled, for. The birth weight threshold of 1.1 kg corresponded to 75% of the average birth weight in the experimental unit, which is also a risk factor for preweaning mortality [17].

## 2. Material and Methods

### 2.1. Ethical Approval

Ethical approval for this study was granted by the Teagasc Animal Ethics Committee (approval no. TAEC133/2016) and was regulated under the HPRA licence (project authorisation no. AE19132/P055). The experiment was carried out in accordance with Irish legislation (SI no. 543/2012) and the EU Directive 2010/63/EU for animal experimentation.

### 2.2. Animals and Management

The study was conducted in the research facilities of Teagasc Moorepark Research Centre, Co. Cork, Ireland, and involved a total of 405 piglets from 97 sows. Genetic background of the piglets was Large White x Duroc. There were 16 primiparous sows and 81 multiparous sows (parity 2:18 sows; parity 3:62 sows; parity 4:1 sow). Piglets were recruited over eight farrowing batches, between February and September 2017.

Piglets were born in conventional farrowing pens (250 × 181 cm), with a sow crate (225 × 60 cm), a heat pad (155 × 37 cm; 2/3 covered) and a water cup and a feeder for piglets. While in the farrowing crate sows had access to a rope (attached to the crate), which was also accessible to the piglets. Sows were assigned to their farrowing crates at approximately day 110 of gestation and induced on their due date if they had not farrowed (D115 of gestation; 2 cc. of Platane^®^, MSD). Piglets were immunised through sow vaccination against mycoplasma hyopneumoniae (Porcilis^®^ M Hyo ID ONCE, MSD), porcine parvovirus (Eryseng^®^ Parvo, HIPRA) and *E. coli* strains (Porcilis^®^ Coliclos, MSD). Experimental piglets were tail-docked at one-day-old following veterinary recommendation, and they received an injection of iron (Gleptosil^®^, Ceva) four days postpartum. Teeth clipping was not performed and the males were not castrated. Piglets were weaned at 27 (S.E.M.: ± 0.1) days old.

Minimum and maximum room temperatures were monitored once daily at 1700 h. Room temperature was maintained around 23 °C around farrowing and decreased by 0.5 °C/week until weaning. The health and vitality of experimental piglets was monitored daily and piglets showing extreme signs of starvation (i.e., not capable of moving, empty belly) by 24 h postpartum were euthanized as per normal farm practice.

### 2.3. Nutrition

Details of the diets provided to sows during gestation (D5 of gestation until farrowing) and lactation (farrowing to weaning) are shown in Table 1. Lactating sows were fed twice a day (i.e., 0900 h and 1500 h) and the amount of feed delivered increased from 2.42 kg on the day of farrowing to 9.10 kg at D28 of lactation (+261 g/d between D0 and D7; +408 g/d between D7 and D14; +164 g/d between D14 and D21; +121 g/d between D21 and D28).

From 10 days postpartum, piglets were provided with creep feed in the farrowing pen. The creep feed was renewed on a little and often basis when the feeder was empty, up to 3 times a day. The creep feed contained 2500 mg/kg Zinc (medicated weaner diet; SCA pre-starter STARTRITE 88 + CINERGY; PROVIMI, UK). Details of the composition of the creep feed are given in Table 2. Intake of creep feed was not recorded in this study.

### 2.4. Experimental Procedure

It was decided to use a single dose of 2 mL fat-based energy based on the commercial product’s instructions. This is similar to previous studies’ protocols [5,22] because this study aimed to be of practical use for the pig industry. Also, the timing of supplementation, at 3 h postpartum, was determined based on the piglets’ energy needs while considering practical aspects for the farmers.

#### 2.4.1. Piglet Recruitment

Piglets were weighed either at birth (Type A) or within 3 h of birth (Type B), and if <1.1 kg were recruited to the study. Time of birth was determined either directly or from video recordings. Once recruited, piglets were returned to the pen at the exact location from where they were picked up, to minimise behavioural disruption and so as not to interfere with normal acquisition of colostrum.

#### 2.4.2. Assignment to Treatments

Upon recruitment, piglets in each litter were assigned to one of four treatments using a predetermined randomisation schedule. This consisted of blocks of four piglets, with treatments assigned at 3 h postpartum in a random order within block. Treatments consisted of dosing with one of three supplements (coconut oil, CO, *n* = 107; commercial product, CP, *n* = 102; water, W, *n* = 100) or sham-dosing (S, *n* = 97). Thus when there were at least four small piglets born, all four treatments were represented in the same litter. When the number of small piglets in a litter exceeded four, subsequent piglets were assigned to the next block. Blocks were left incomplete within a litter if the number of small piglets was not a multiple of four. Treatments were balanced between genders, and the overall gender ratio (M:F) was 0.9.

All experimental piglets were handled similarly. At 3 h postpartum they were picked-up by placing a hand under their belly, lifted from the ground and maintained alongside the experimenter’s chest. The experimenter held a syringe containing 2 mL of supplement and gently squeezed the contents into the piglet’s mouth. When the piglet ingested the entire dose, it was gently released in the pen. Sham-dosed piglets were handled in the same way as other treatments but the syringe was empty.

#### 2.4.3. Energy Supplement Products

Table 2 summarises the (estimated) contents of the commercial product, coconut oil and sow colostrum. All three supplements (i.e., water, coconut oil and the commercial product) were placed on a heat pad at least 30 min prior to dosing, to ensure that the coconut oil remained liquid and that all supplements were of the same temperature when administered to piglets.

### 2.5. Measurements

#### 2.5.1. Health and Mortality

Any incidence of disease or death was recorded daily throughout the experiment. Cause of death was identified and recorded.

#### 2.5.2. Growth

Piglets were weighed at birth, at 3 h postpartum (i.e., assignment to treatment, D0), 27 h postpartum (i.e., 24 h after treatment, D1) and then on at D5, D7, D10, D14, D21 and at weaning.

#### 2.5.3. IUGR Score

Upon recruitment, piglets were scored for IUGR according to the shape of their head (0 = normal, 1 = ‘dolphin’ shape), presence or absence of bulging eyes (no = 0, yes = 1) and wrinkles around the nose (no = 0, yes = 1) [23]. The three scores were then summed, so that piglets presenting none of the characteristics were considered “normal” (overall score = 0). The presence of one (score 1, “mild-IUGR”) to three (score 3, “severe-IUGR”) was indicative of increasing levels of IUGR.

#### 2.5.4. Piglet Vitality

To test the vitality of piglets at birth, a simple standardised test suitable for use by stock personnel to identify piglets ‘at risk’ was developed. The piglets were tested at assignment to treatment (i.e., 3 h postpartum) and 24 h after (i.e., 27 h postpartum, D1) using the following procedure. The piglet was lifted from the ground by placing one or both hands under the belly of the piglet and placed gently on the passageway floor (solid plastic) while the stopwatch was started. The initial position of the piglet was scored (1: piglet standing on its four legs; 0: piglet sitting or lying). Whether or not the piglet moved within 10 s after being placed on the floor was then recorded; a score of 1 was given if the piglet stood up (if sitting/lying) or walked in the passageway (if standing up), and score 0 was given if the piglet did not move from its initial position. Scores were then added to obtain an overall vitality score (ranging from 0 = weak piglet to 2 = vigorous piglet).

#### 2.5.5. Blood Glucose Level

This was measured at assignment to treatment (i.e., 3 h postpartum) for a subset of piglets (CO = 45 piglets, CP = 38 piglets, W = 49 piglets and S = 44 piglets) and 24 h after treatment application (i.e., 27 h postpartum) for all piglets using a hand held device (iDIA Blood Glucose monitor, Arctic Medical). Blood samples were obtained by pricking the ear vein with a small lance (provided with the kit; 30G/0.3 mm) so that a small drop of blood emerged (approximately 2–3 mm in diameter). A reading from the device was obtained by simply touching the analytical strip against the drop of blood. 

#### 2.5.6. Rectal Temperature

Rectal temperature of piglets was measured at 3 h postpartum (i.e., assignment to treatment) and 1 h after treatment application using a digital thermometer (VedoFamily, Pic Solution, Italy).

### 2.6. Statistical Analysis 

Statistical analysis was performed using SAS 9.4 (SAS Inst. Inc., Cary, NC). The experimental unit for the analysis was the pig nested within litter. General Linear Models (GLM; PROC MIXED) and Generalized Linear Mixed Models (GLMM; PROC GLIMMIX) were fitted by Residual Pseudo Likelihood approximation method for models of (non-)normal data, with appropriate link functions and error structures depending on the nature of the response variable. Estimated Least Square Means, or their back-transformed values, are reported. Statistically significant terms were determined when *p* < 0.05 and tendencies were considered when the *p*-value lay between 0.05 and 0.1. Treatment, gender and IUGR score were included in all models as fixed effects. The effects of the interactions between IUGR score and treatment and of the interaction between gender and treatment were investigated but there was no effect for any variable, and thus the interactions were not included in the final models. Initially the number of pigs in the litter was included in the model as a covariate, but only retained if significant. Sow and replicate were included as random effects in all models. Contrast statements were used to investigate differences between treatments which contained fat-based energy (CO and CP) and treatments which did not (S and W). As none of these comparisons were significant they were not reported. Contrast statements were also used to investigate differences between one treatment and the other three, where numerical differences suggested that there may be significant differences (e.g., blood glucose concentration).

Mortality rates at 24 h and preweaning were analysed using GLMM with binary distributions and logit link function. Vitality scores were analysed using GLMM with Poisson distribution and log link function.

All other variables (i.e., weight, growth, blood glucose content and temperature) were analysed using GLMs which included the initial measure as a covariate. Day was included as a fixed effect in models analysing piglet weight during lactation, and the repeated effect of day within pig was also accounted for. There were less subjects at the blood collection at 3 h postpartum (assignment to treatments) than in the second collection (27 h postpartum). The calculation of the change during the 24 h after assignment only included the subjects that had two values, thus it is different than subtracting the estimated value of blood glucose content at 3 h postpartum from the estimated value at 27 h.

## 3. Results

### 3.1. Health and Mortality

**Figure 1:**
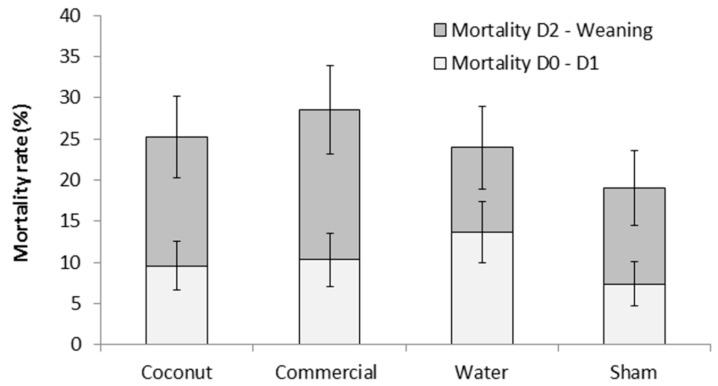
Mortality rates (%) of piglets during the first 24 h postpartum (D0–D1) and until weaning (D2–Weaning). Piglets were either given a 2 mL oral supplementation at 3 h postpartum (coconut oil, commercial product or water) or were sham-dosed. Preweaning mortality rate is the addition of light and dark grey bars. No significant difference was detected.

**Figure 2:**
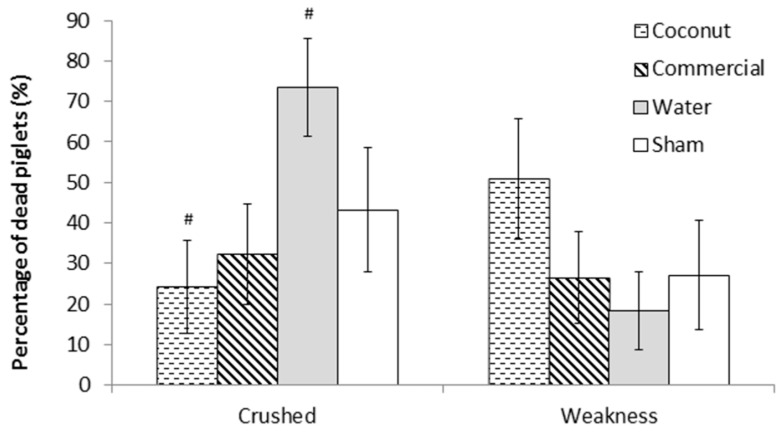
Percentage of dead piglets per main cause of mortality (crushing or weakness). Piglets were either given a 2 mL oral supplementation at 3 h postpartum (Coconut oil, commercial product or water) or were sham-dosed. Tendency for difference between treatments (*p* = 0.06) is shown by #.

There was no effect of treatment on piglet mortality either to 24 h or weaning (F_3,303_ = 0.78 and F_3,303_ = 0.76, respectively; *p* > 0.5; Figure 1). Mortality rates at 24 h ranged from 7.4 ± 2.69% (S piglets) to 13.7 ± 3.73% (W piglets) and preweaning mortality rates ranged from 19.0 ± 4.57% (S piglets) to 28.5 ± 5.40% (CP piglets). However, there was a tendency for an effect of treatment on the incidence of crushing (F_3,44_ = 2.68; = 0.06; Figure 2). Indeed, piglets that received a dose of water tended to have a higher incidence of crushing compared to piglets that received a dose of coconut oil (73.4 ± 12.09% vs. 24.3 ± 11.49%; t_44_ = −2.66; *p* = 0.05) and piglets that received a dose of commercial product (73.4 ± 12.09% vs. 32.2 ± 12.37%; t_44_ = −2.34; *p* = 0.06).

### 3.2. Piglet Growth

**Figure 3:**
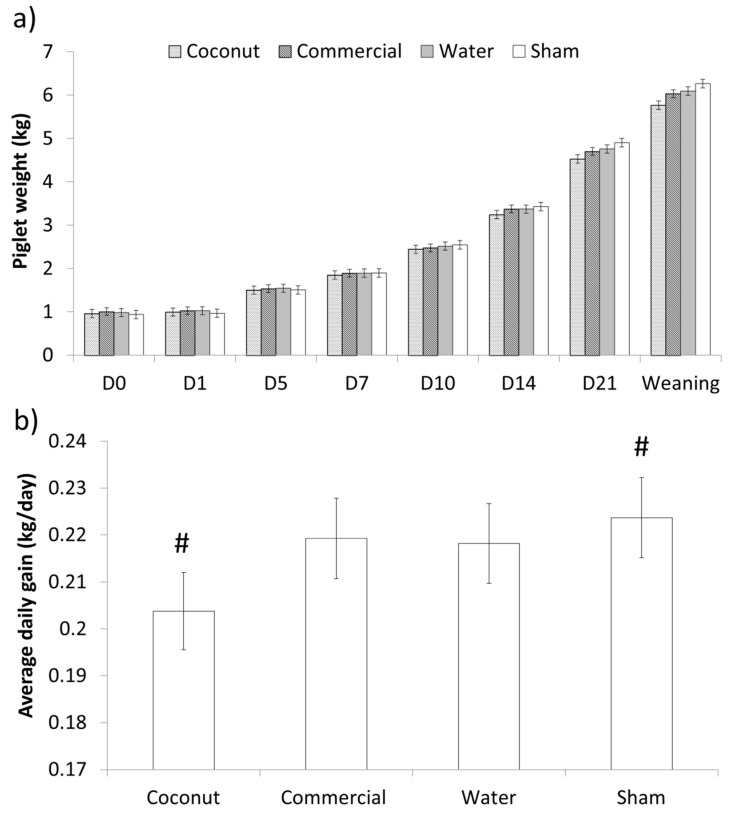
Preweaning weights (**a**) and average daily gain (**b**) of piglets born under 1.1 kg. Piglets were either given a 2 mL oral supplementation at 3 h postpartum (Coconut oil, commercial product or water) or were sham-dosed. Tendency for difference between treatments (*p* = 0.07) is shown by #.

There was no effect of treatment on the weight of piglets at supplementation or 24 h after (*p* > 0.70 and *p* > 0.10, respectively; Table 3). Additionally, average daily gain (ADG) did not differ between treatments during the 24 h following supplementation (*p* > 0.20; Table 3). Weights did not differ over the entire lactation period (F_21,214_ = 1.32; *p* > 0.10; Figure 3). However, preweaning ADG tended to differ between treatments (F_3,259_ = 2.38; *p* = 0.07), as sham-dosed piglets tended to have a higher ADG than piglets given coconut oil (Figure 3).

### 3.3. IUGR Score

The mean IUGR score attributed to piglets at birth did not differ between treatments (S: 1.68 ± 0.151, CO: 1.54 ± 0.137, CP: 1.66 ± 0.147, W: 1.65 ± 0.147; F_3,401_ = 0.26; *p* > 0.80). However, within each IUGR score there were differences on the percentage of piglets attributed this score within treatment (Table 3). There was no difference on the percentage of piglets attributed an IUGR score of 0 (F_3,400_ = 1.4; *p* > 0.2) or a score of 3 (F_3,400_ = 1.14; *p* > 0.3) across treatments. However, the percentage of piglets attributed an IUGR score of 2 (F_3,400_ = 3.80; *p* < 0.05) differed between treatments, and there was a tendency for the percentage of piglets assigned an IUGR score of 1 to differ (F_3,400_ = 2.40; *p* = 0.07). There was a lower percentage of piglets with an IUGR score of 2 in the S treatment group than in the CP and W groups (S: 21.1 ± 4.33 vs. CP: 41.6 ± 5.25, and W: 38.6 ± 5.24; t_400_ = −3.06 and t_400_ = −2.64, respectively; *p* < 0.05).

### 3.4. Vitality Score

There was no effect of treatment on piglet vitality scores either at or 24h after supplementation (*p* > 0.80 and *p* > 0.90, respectively; Table 3). The change in vitality score between supplementation and 24 h after was not different between treatments (*p* > 0.80; Table 3).

### 3.5. Blood Glucose Level

**Figure 4:**
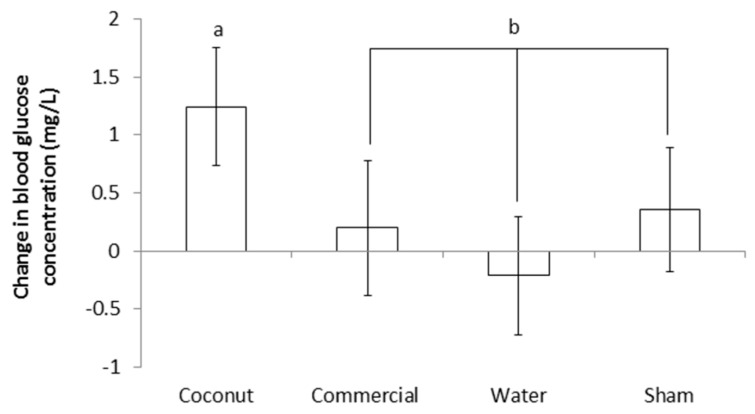
Change in blood glucose concentration during the 24 h following assignment to treatments (i.e., at 3 h postpartum). Piglets were either given a 2 mL oral supplementation at 3 h postpartum (coconut oil, commercial product or water) or were sham-dosed. Different superscript letters (a and b) (*p* < 0.05).

Blood glucose level did not differ between treatments at supplementation or at 24 h after supplementation (*p* > 0.50 and *p* > 0.80, respectively; Table 3). The change in blood glucose concentration during the 24 h following supplementation was not significantly different between treatments (*p* > 0.10; Table 3). However, CO piglets had a numerically higher increase in blood glucose content, and the difference between CO piglets and the three other treatments together (i.e., CP, W and S) was significant (F_1,130_ = 4.21; *p* < 0.05; Figure 4).

### 3.6. Rectal Temperature

Rectal temperature at supplementation and 1 h after supplementation did not differ between treatments (*p* > 0.40 and *p* > 0.80, respectively; Table 3). There was no treatment effect on the change in temperature during the hour following supplementation (*p* > 0.40; Table 3).

## 4. Discussion

The present study investigated the effect of fat-based energy supplementation to neonatal piglets of low birth weight (i.e., <1.1 kg birth weight) on their survival, growth and vitality, as it could be applied on commercial farms. Thus the timing of treatment administration was set to 3 h postpartum because farmers are more likely to manage the litters (e.g., do split-suckling, cross-fostering, etc.) when sows have finished farrowing. The results suggest that a single administration of 2 mL of fat-based energy at 3 h postpartum did not improve any of the parameters measured. However, the increase in blood glucose concentration was greater in piglets supplemented with coconut oil, compared to the other three treatments together.

In line with the results of Lepine et al. [14], coconut oil supplementation was associated with an increase in blood glucose concentration at 24 h, but did not improve piglet survival. This was the case even though the amount of medium-chain triglycerides (MCT) supplemented in our study, as part of the coconut oil composition (Table 1; approximately 60% of coconut oil = 1.2 mL/dose) was twenty times lower than that supplemented by Lepine and colleagues [14] (25 mL, 464.4 KJ). These findings question the validity of using blood glucose concentration as an indicator of survival in the case of energy supplementation, moreover if the time and duration of suckling are not recorded. Coconut oil and the commercial product were rich in MCT, and thus MCFA, which may have provided the piglets in both studies with a feeling of satiety [14,24], and thus reduced their appetite. The effect of several doses of MCT on suckling behaviour should be investigated in order to determine if MCT/MCFA is suitable for energy supplementation of neonatal piglets, and if so, which quantity is most beneficial.

While blood glucose content of piglets supplemented with coconut oil numerically had the greatest increase after supplementation, the blood glucose content of piglets supplemented with water was the only one to decrease. Also, piglets in the latter treatment tended to have a higher mortality from crushing than piglets supplemented with coconut oil. This suggests a detrimental effect of filling piglets’ stomach with energy-free liquids. This may have given them a feeling of satiety which could have prevented their consumption or normal absorption of colostrum. Further research on gastric capacity and feelings of satiety in low birth weight piglets is warranted, so that nutritional interventions can be optimised.

In contrast to our results, however, similar studies reported that neonatal energy supplementation using other fats effectively reduces the mortality of small piglets [5,25]. These two studies used similar doses of energy products (3 g in Declerck et al. [5]; 2 × 1 mL and 5 mL in Muns et al. [25]). In the study of Declerck et al. [5], piglets born under 1.00 kg and energy supplemented had a lower mortality rate than nonsupplemented piglets (i.e., until D3, D7 and D21). However, piglets with a birth weight between 1 kg and 1.2 kg that received supplementation tended to have a higher mortality rate than control piglets on D3. This suggests that the energy supplementation primarily benefits very low birth weight piglets. However, in the above mentioned studies all piglets within a litter received the same treatment and were kept together on the same sow, whereas in the present study piglets were randomly assigned to the different treatments within the same litter. Thus we could account for the effect of sow (e.g., maternal abilities) on the variables measured. Muns and colleagues [11] found differences in the effectiveness of fat-based energy supplementation between litters reared by primiparous or multiparous sows. Our study design better controlled the potential impact of the sows’ maternal abilities, which is important when considering piglet survival (e.g., crushing [26]) and growth (e.g., nursing frequency [27]).

Additionally, piglet IUGR level, which may influence the response to energy supplementation, was not considered in previous studies. Not all low birth weight piglets are growth retarded, and whether or not they are could impact their response to energy supplementation. For instance, IUGR piglets had lower body temperature and lower blood plasma glucose content compared to normal piglets [28]. In the present study, IUGR score was included in all statistical models, and it had a significant effect on some of the variables of interest. The interaction between IUGR score and treatment was not significant for any variable, which may suggest that piglets with differing degrees of growth retardation did not react differently. However, since the distribution of piglets between IUGR scores differed amongst treatments, the present study could not adequately address this issue, and a more controlled study is warranted to draw stronger conclusions.

How much, what, when and the way an energy supplement is administered can be important sources of variations between studies. It could be considered that the quantity of the fat-based energy supplements administrated to piglets in the present study was insufficient to have had any positive effect. We estimated that 2 mL of either of our energy supplements should have given piglets the capacity to produce heat for up to 7 h (calculations based on products energy value and piglet energy consumption from Mellor and Cockburn [1]). An (underestimated) average colostrum intake of 300 g per piglet was reported [29], which is the equivalent of an energy intake of approximately 2010 KJ within 24 h (energy level of colostrum = 670 KJ/100 g [18]). Thus the energy supplementation we provided supplied only 3.5% (71 KJ in CP) to 3.7% (74 KJ in CO) of the energy normally ingested by neonatal piglets during the first 24h. This relatively low contribution could explain why there were no differences in rectal temperature or survival at 24 h postpartum. Therefore, in further studies, the amount of energy supplied to neonatal piglets should be superior to that of the present study. Evaluation of the energy requirements of piglets have focused on heat production [1,30,31,32]. However, energy is also utilised by piglets for movement in the pen, stimulating the udder and competing for a teat. Therefore, energy expenditure by the piglet is likely significantly higher than the estimation, and will vary according to the layout of the farrowing pen and how supplementary heat is provided (heat lamps, heat pads, etc.). Floor type and bedding, as well as the ambient temperature in the room (and consequently the time of the day and season), could also affect energy expenditure. This knowledge gap highlights the need to improve knowledge about the energy needs of piglets at birth.

The composition of the product administered to the piglets is important when comparing results between studies. For instance, the energy contribution varies between studies, and some commercial products include immunoglobulins in their composition, which seems to increase IgG levels in small piglets but do not enhance their survival [25]. Furthermore, it is suggested that low birth weight piglets have a reduced energy metabolism, probably because of a lower villus size and consequentially a reduced intestinal surface area available for nutrient absorption [33], which could be reversed by arginine supplementation [34].

Modern pig breeds have been selected for lean meat, and thus low body fat, thus piglets have lower thermoregulation abilities and depend more on colostrum ingestion than breeds with high body fat (e.g., Meishan pigs [35]). Therefore, the timing of supplementation may be crucial in modern breeds. In the present study, it was decided that administrating energy to piglets at 3 h postpartum was the most practical timing. First, farmers are more likely to care for the litter within a few hours after birth. Second, colostrum intake may be hindered if supplementation occurs before the piglets have suckled, while supplementing low birth weight piglets after 3 h postpartum might put them in danger of complete depletion of their body energy reserves. Finally, it has been estimated that low birth weight piglets are only able to sustain heat production for ~3 h [1], so providing energy before or at this time point might help them recover from heat loss. It is not impossible that administrating an energy dose earlier than at 3 h postpartum would result in a greater (or at least detectable) increase rectal temperature, since piglets experience a temperature drop shortly after birth [36]. However, there was no relationship between colostrum intake and thermal status on neonatal piglets at 2 h postpartum [37], suggesting that heat preservation (through huddling and closeness to heat sources) rather than energy intake was important for piglets’ thermoregulation success. Recent work by Muns and colleagues [25] found that supplementing light piglets (i.e., <1.35 kg birth weight) with commercial products (i.e., 1 mL of Lianol Colostro or 5 mL of ColoBoost) twice within 12 h postpartum was effective in improving their survival at 24 hours, but not in improving their growth. However, dosing the piglets twice within 12 h postpartum is likely to be challenging on commercial farms, especially when sows farrow during the night.

Finally, another possible reason for not detecting differences between treatments in the current study may be that the experiment was conducted in a new research facility (1 year old) where standards of pig health and hygiene were high. For instance, since vaccination of piglets was limited to the day of weaning, handling of piglets during lactation was reduced compared to on commercial pig farms and thus there was a lower risk of disease transmission across farrowing pens. Therefore, it is possible that piglets were not challenged to the same magnitude as piglets on commercial farms.

## 5. Conclusions

In conclusion, a single dose of fat-based energy boost at three hours following birth did not improve the survival of low birth weight piglets, their body temperature or growth rate. However, our results suggest that further work should take into account the piglets’ level of IUGR when evaluating their energy needs and how to fulfil them. The use of coconut oil as a fat-based energy supplement might deserve further attention as it increased the blood glucose concentration of low birth weight piglets.

## Figures and Tables

**Table 1:** Ingredient composition and chemical analysis of sows’ gestation and lactation diets and piglets’ creep feed.

Diet Analysis	Gestation Diet ^1^	Lactation Diet ^2^	Creep Feed ^3^
Ingredient composition (%)			
Wheat	-	44.35	-
Barley	75.30	30.00	-
Soyahulls	12.18	-	-
Soya	8.96	16.00	-
Soya oil	1.10	6.44	-
Limestone flour	0.91	1.15	-
Mono Dicalcium Phosphate	0.65	0.84	-
Salt	0.40	0.40	-
Lysine HCl (78.8%)	0.22	0.44	-
Vitamin and trace minerals ^4^	0.15	0.15	-
L-Threonine (98%)	0.06	0.14	-
DL-Methionine	0.06	0.06	-
L-Tryptophan		0.02	-
Natuphos 5000 FTU/g ^5^	0.01	0.01	-
Chemical analysis (g/kg)			
Dry matter	873.27	876.96	895.00
Crude protein	140.00	157.57	205.50
Ash	47.34	48.31	59.50
Crude fat	31.44	79.76	70.50
Crude fibre	80.00	33.32	24.50
Sugar	25.82	31.09	-
Starch	399.94	422.66	-
Neutral Detergent Fibre	213.41	122.73	-
Acid Detergent Fibre	108.59	43.53	-
Digestible energy (MJ/kg)	13.20	15.10	-
Lysine	8.20	10.80	15.50
Methionine	2.70	3.00	5.90
Threonine	5.50	6.90	-
Tryptophan	1.70	-	-
Ca	7.20	8.10	5.30
P	5.00	5.50	6.20

^1^ This diet was given to gestating sows from D5 to D115 of gestation (farrowing). ^2^ This diet was given to lactating sows from farrowing to weaning (approximately 28 days). ^3^ This diet was given to suckling piglets from D10 postpartum until weaning. Vitamins: 3a700 Vitamin E: 14985 IU; 3a672a Vitamin A: 13490 IU; E671 Vitamin D3: 2700 IU. Additives: Endo-1,4,-Beta-Xylanase: 100 IUA; E321 BHA/ethoxyquin antioxidants: 5 mg; Proviox50: 100 ppm. 4. Vitamin–mineral premix provided per kg of complete diet: Cu, 15 mg; Fe, 70 mg; Mn, 62 mg; Zn, 80 mg; I, 0.6 mg; Se, 0.2 mg; vitamin A, 10000IU; vitamin D3, 1000IU; vitamin E, 100IU; vitamin K, 2 mg; vitamin B12, 15 mg; riboflavin, 5 mg; nicotinic acid, 12 mg; pantothenic acid, 10 mg; choline chloride, 500 mg; biotin, 200 mg; folic acid, 5 mg; vitamin B1, 2 mg; vitamin B6, 3 mg. 5. Diets contained 500 FTU phytase per kg finished feed from Natuphos 5000 (BASF, Ludwigshafen, Germany).

**Table 2:** Details of the composition of the two energy supplements (coconut oil and commercial product) used in the study and of sow colostrum [18] for comparison. Unless stated otherwise values are in percentage of total composition.

Product	Sow Colostrum ^1^	Coconut Oil ^2^	Commercial Product ^3^
Dose recommended	200 g/24 h	-	2 × (2 mL/24 h)
Coconut content (%)	0	100	15
Calories for 2 mL	13.4 kJ	72 kJ	71 kJ
Chemical analysis (%)			
Fat	6.50	100.00	100.00
Fatty acids profile (% fat)			
Caproic acid C6:0	-	0.50	1.40
Caprylic acid C8:0	-	7.30	44.00
Capric acid C10:0	0.40	6.10	24.00
Lauric acid C12:0	0.50	47.00	8.80
Myristic acid C14:0	3.20	18.30	3.60
Palmitic acid C16:0	32.20	9.00	2.80
Stearic acid C18:0	6.40	3.40	-
Oleic acid C18:1	38.50	6.60	3.60
Linoleic acid C18:2	12.70	2.20	7.20
Linolenic acid C18:3	0.80	0.00	1.00
Lactose	3.40	0.00	0.00
Protein	12.30	0.00	0.00
Fibre	-	-	-
Ash	0.70	-	2.50
Water	75.00	0.00	2.00
Vitamins (µg/mL)			
Vitamin A	1.14	0.00	1250.00
Vitamin D	0.015	-	37.50
Vitamin C	190.00	0.00	-
Vitamin E	10.00	90.00	10000.00
Iron (µg/mL)	2.84	40.00	-
Magnesium (µg/mL)	104.00	0.00	0.00
Calcium (µg/mL)	800.00	0.00	-
Phosphorus (µg/mL)	1080.00	-	0.00

^1^ From Hurley et al. [18], fatty acid profile from Csapo et al. [19]; ^2^ Means calculated [20,21,22]; ^3^ From company brochure.

**Table 3:** Mean (±SE) values for the variables measured (intra-uterine growth retardation (IUGR) score, weight, rectal temperature, blood glucose content and vitality score (0–2)) at the time of supplementation (3 h postpartum) and 1 h (rectal temperature only) and 24 h postsupplementation. Piglets were either given a 2 mL oral supplementation at 3 h postpartum (coconut oil, commercial product or water) or were sham-dosed. Different superscript letters (a and b) *p* < 0.05.

Measurements	Time Postpartum	Coconut Oil	Commercial Product	Water	Sham-Dosed	F-Value	*p*-Value
**Percentage of IUGR piglets (%)**							
Score 0	-	16.5 (±4.64)	12.5 (±4.03)	10.7 (±3.75)	7.3 (±2.93)	F_3,400_ = 1.14	N.S.
Score 1	-	29.9 (±5.32)	23.9 (±4.93)	29.2 (±5.44)	41.4 (±6.07)	F_3,400_ = 2.40	0.07
Score 2	-	29.6 (±4.7)	41.6 (±5.25) ^a^	38.6 (±5.24) ^a^	21.1 (±4.34) ^b^	F_3,400_ = 3.80	<0.05
Score 3	-	19.7 (±4.72)	18.1 (±4.62)	18 (±4.59)	27.4 (±5.77)	F_3,400_ = 1.40	N.S.
**Supplementation (1)**							
weight (kg)	3 h	0.92 (±0.008)	0.91 (±0.008)	0.91 (±0.008)	0.91 (±0.008)	F_3,375_ = 0.54	N.S
temperature (˚C)	3 h	37.5 (±0.31)	37.7 (±0.32)	37.5 (±0.31)	37.7 (±0.32)	F_3,389_ = 0.8	N.S
glucose (mg/L)	3 h	3.56 (±0.393)	3.54 (±0.431)	3.99 (±0.385)	4.1 0(±0.411)	F_3,137_ = 0.64	N.S
vitality score	3 h	1.7 (±0.13)	1.8 (±0.14)	1.7 (±0.13)	1.8 (±0.14)	F_3,1_ = 0.21	N.S
**Postsupplementation (2)**							
weight (kg)	27 h	0.95 (±0.019)	0.94 (±0.019)	0.96 (±0.019)	0.94 (±0.020)	F_3,332_ = 2.1	N.S
temperature (˚C)	4 h	37.7 (±0.09)	37.6 (±0.09)	37.7 (±0.09)	37.7 (±0.09)	F_3,382_ = 0.31	N.S
glucose (mg/L)	27 h	3.79 (±0.245)	3.55 (±0.261)	3.67 (±0.27)	3.57 (±0.267)	F_3,268_ = 0.25	N.S
vitality score	27 h	1.8 (±0.14)	1.8 (±0.14)	1.8 (±0.15)	1.8 (±0.15)	F_3,1_ = 0.03	N.S
**Change (2-1)**							
weight (g)	-	28.3 (±7.14)	16.1 (±7.48)	33.7 (±7.81)	23.3 (±7.64)	F_3,291_ = 1.36	N.S
temperature (˚C)	-	0.5 (±0.09)	0.4 (±0.10)	0.5 (±0.10)	0.4 (±0.10)	F_3,340_ = 0.90	N.S
glucose (mg/L)	-	1.24 (±0.506)	0.20 (±0.577)	−0.21 (±0.506)	0.36 (±0.533)	F_3,131_ = 1.75	N.S
vitality score	-	0.1 (±0.05)	0.1 (±0.05)	0.2 (±0.08)	0.2 (±0.06)	F_3,319_ = 1.04	N.S
